# Supplementary material for: Patients with Metachronous Peritoneal Metastatic Mucinous Colorectal Adenocarcinoma Benefit More from Cytoreductive Surgery (CRS) and Hyperthermic Intraperitoneal Chemotherapy (HIPEC) than Their Synchronous Counterparts
Source: Cancers (Basel). 2022 Aug 17;14(16):3978. doi: 10.3390/cancers14163978 (PMC9406628; doi:10.3390/cancers14163978)
Supplement: Supplementary file 1 [file cancers-14-03978-s001.zip › cancers-1866884-supplementary.pdf]

Table S1. Complete pre-, peri- and postoperative demographic and clinical characteristics of study participants having synchronous peritoneal metastases (PM). Unit of frequency and survival data is the number of observations (percentage) and median survival time (95% confidence interval), respectively.

Clinical Characteristics	Synchronous PM (*n* = 129)	sAC (*n* = 93)	sMAC (*n* = 36)	Crude *p*-value	Adjusted *p*-value
Age (year)	54.23 ± 12.14	54.74 ± 11.09	52.90 ± 14.60		1.0000
Body mass index (kg/m²)	25.27 ± 4.86	25.52 ± 5.07	24.66 ± 4.32		1.0000
Sex (male:female)	63:66 (48.8%:51.2%)	44:49 (47.3%:52.7%)	19:17 (52.8%:47.2%)		1.0000
ASA score					1.0000
- I–II	33 (25.6%)	21 (22.6%)	12 (33.3%)		
- III–IV	96 (74.4%)	72 (77.4%)	24 (66.7%)		
Tumor location					1.0000
- Cecum	34 (26.4%)	23 (24.7%)	11 (30.6%)		
- Ascending colon	28 (21.7%)	17 (18.3%)	11 (30.6%)		
- Hepatic flexure	7 (5.4%)	6 (6.5%)	1 (2.8%)		
- Transverse colon	5 (3.9%)	3 (3.2%)	2 (5.6%)		
- Splenic flexure	2 (1.6%)	1 (1.1%)	1 (2.8%)		
- Descending colon	8 (6.2%)	7 (7.5%)	1 (2.8%)		
- Sigmoid colon	30 (23.3%)	23 (24.7%)	7 (19.4%)		
- Rectosigmoid	4 (3.1%)	3 (3.2%)	1 (2.8%)		
- Rectum	9 (7.0%)	8 (8.6%)	1 (2.8%)		
- Multiplex	2 (1.6%)	2 (2.2%)	0 (0%)		
Sidedness					1.0000
- Left-sided	44 (34.1%)	34 (36.6%)	10 (27.8%)		
- Right-sided	74 (57.4%)	49 (52.7%)	25 (69.4%)		
- Rectum	9 (7.0%)	8 (8.6%)	1 (2.8%)		
- Both (multiplex)	2 (1.6%)	2 (2.2%)	0 (0%)		
Stage T (size of tumor) [1]				**0.0518**	1.0000
- 1-2	3 (2.3%)	3 (3.2%)	0 (0%)		
- 3	31 (24.0%)	27 (29.0%)	4 (11.1%)		
- 4	93 (72.1%)	62 (66.7%)	31 (86.1%)		
Stage N (lymph node metastases) [1]				**0.0343**	1.0000
- 0	23 (17.8%)	14 (15.1%)	9 (25.0%)		
- 1	33 (25.6%)	29 (31.2%)	4 (11.1%)		
- 2-3	71 (55.0%)	48 (51.6%)	23 (63.9%)		
RAS (wild:mutant) [2]	24:26	21:17	3:9	–	–
BRAF (wild:mutant) [2]	14:4	12:3	2:1	–	–
Microsatellite (stable:instable) [2]	32:2	30:1	2:1	–	–
Lineage of chemotherapy				**0.0201**	1.0000
- First line	90 (69.8%)	70 (75.3%)	20 (55.6%)		
- Second line	8 (6.2%)	7 (7.5%)	1 (2.8%)		
- Third line	3 (2.3%)	1 (1.1%)	2 (5.6%)		
Usage of biological agents					1.0000
- Bevacizumab	32 (24.8%)	21 (22.6%)	11 (30.6%)		
- Cetuximab / Panitumumab	26 (20.2%)	22 (23.7%)	4 (11.1%)		
Radiotherapy	8 (6.2%)	6 (6.5%)	2 (5.6%)		1.0000
Any previous surgeries related to CRC [3]					
- Number of surgeries (single:multiple)	69:41 (53.5%:31.8%)	48:34 (51.6%:36.6%)	21:7 (58.3%:19.4%)		1.0000
- Primary tumor resecated	93 (72.1%)	70 (75.3%)	23 (63.9%)		1.0000

Type of previous surgeries [3]					
- Primary tumor resection only	45 (34.9%)	31 (33.3%)	14 (38.9%)		1.0000
- Laparoscopic exploration only	11 (8.5%)	7 (7.5%)	4 (11.1%)		1.0000
- Metastasis removal (excl. Perit.)	43 (33.3%)	34 (36.6%)	9 (25.0%)		1.0000
- Partial peritonectomy	30 (23.3%)	27 (29.0%)	3 (8.3%)	**0.0114**	0.6959
Peritoneal carcinomatosis index	7.67 ± 6.63	6.69 ± 5.83	10.22 ± 7.89	**0.0080**	0.4983
CC score					
- CC-0	112 (86.8%)	83 (89.2%)	29 (80.6%)		1.0000
- CC-1	10 (7.8%)	7 (7.5%)	3 (8.3%)		
- CC-2	7 (5.4%)	3 (3.2%)	4 (11.1%)		
Time between CRS+HIPEC and tumor diagnosis (month)	10.06 ± 10.24	10.10 ± 8.41	9.97 ± 14.04		1.0000
HIPEC medication					
- Oxaliplatin	77 (59.7%)	56 (60.2%)	21 (58.3%)		1.0000
- Mitomycin	52 (40.3%)	37 (39.8%)	15 (41.7%)		
Oxaliplatin (mg)	302.58 ± 15.93	301.77 ± 13.23	304.76 ± 21.82		1.0000
Mitomycin (mg)	28.17 ± 8.11	27.16 ± 6.51	30.67 ± 11.00		1.0000
Temperature of HIPEC					
- 41°C	13 (10.1%)	11 (11.8%)	2 (5.6%)		1.0000
- 42°C	116 (89.9%)	82 (88.2%)	34 (94.4%)		
Duration of HIPEC (min)					
- 30	75 (58.1%)	55 (59.1%)	20 (55.6%)		1.0000
- 60	15 (11.6%)	9 (9.7%)	6 (16.7%)		
- 90	39 (30.2%)	29 (31.2%)	10 (27.8%)		
Length of surgery excl. HIPEC (min)	269 ± 105.5	264 ± 104.5	282 ± 108.4		1.0000
Surgical procedures					
- Perit.: parietal	83 (64.3%)	58 (62.4%)	25 (69.4%)		1.0000
- Perit.: pelvis	78 (60.5%)	57 (61.3%)	21 (58.3%)		1.0000
- Perit.: omental bursa	10 (7.8%)	4 (4.3%)	6 (16.7%)	**0.0281**	1.0000
- Perit.: right upper quadrant	38 (29.5%)	23 (24.7%)	15 (41.7%)	**0.0840**	1.0000
- Perit.: left upper quadrant	21 (16.3%)	9 (9.7%)	12 (33.3%)	**0.0025**	0.1585
- Ana.: small bowel–small bowel	27 (20.9%)	22 (23.7%)	5 (13.9%)		1.0000
- Ana.: stomach–small bowel	1 (0.8%)	0 (0%)	1 (2.8%)		1.0000
- Ana.: small bowel–colon	36 (27.9%)	24 (25.8%)	12 (33.3%)		1.0000
- Ana.: colon–colon	7 (5.4%)	7 (7.5%)	0 (0%)		1.0000
- Ana.: colon–rectum	54 (41.9%)	36 (38.7%)	18 (50.0%)		1.0000
- Ana.: small bowel–rectum	5 (3.9%)	2 (2.2%)	3 (8.3%)		1.0000
- Colostomy	2 (1.6%)	1 (1.1%)	1 (2.8%)		1.0000
- Ileostomy	26 (20.2%)	20 (21.5%)	6 (16.7%)		1.0000
- Colon resection	60 (46.5%)	39 (41.9%)	21 (58.3%)		1.0000
- Small bowel resection	43 (33.3%)	28 (30.1%)	15 (41.7%)		1.0000
- Rectosigmoid resection	46 (35.7%)	31 (33.3%)	15 (41.7%)		1.0000
- Splenectomy	9 (7.0%)	3 (3.2%)	6 (16.7%)	**0.0142**	0.8531
- Cholecystectomy	61 (47.3%)	44 (47.3%)	17 (47.2%)		1.0000
- Bladder resection	2 (1.6%)	2 (2.2%)	0 (0%)		1.0000
- Greater omentectomy	87 (67.4%)	63 (67.7%)	24 (66.7%)		1.0000
- Lesser omentectomy	38 (29.5%)	26 (28.0%)	12 (33.3%)		1.0000
- Hepatic metastasectomy	24 (18.6%)	20 (21.5%)	4 (11.1%)		1.0000
- No. of resecated hepatic metastases	0.26 ± 0.74	0.32 ± 0.85	0.08 ± 0.28		1.0000

Surgical procedures					
- Stomach resection	2 (1.6%)	0 (0%)	2 (5.6%)		1.0000
- Hysterectomy	28 (21.7%)	19 (20.4%)	9 (25.0%)		1.0000
- Adnexectomy	34 (26.4%)	26 (28.0%)	8 (22.2%)		1.0000
- Resection of other organs	31 (24.0%)	23 (24.7%)	8 (22.2%)		1.0000
Length of hospital stay (day)	16.83 ± 12.24	16.68 ± 12.45	17.22 ± 11.85		1.0000
Length of ICU stay (day)	4.11 ± 2.98	4.17 ± 3.36	3.94 ± 1.69		1.0000
Complication grade (Clavien–Dindo)					
- I	71 (55.0%)	52 (55.9%)	19 (52.8%)		
- II	33 (25.6%)	21 (22.6%)	12 (33.3%)		
- III	22 (17.1%)	18 (19.4%)	4 (11.1%)		1.0000
- IV	2 (1.6%)	1 (1.1%)	1 (2.8%)		
- V	1 (0.8%)	1 (1.1%)	0 (0%)		
Complications					
- Anastomotic insufficiency/leak	6 (4.7%)	4 (4.3%)	2 (5.6%)		1.0000
- Pancreatitis	1 (0.8%)	0 (0%)	1 (2.8%)		1.0000
- Fistula	1 (0.8%)	0 (0%)	1 (2.8%)		1.0000
- Pulmonary embolism	2 (1.6%)	2 (2.2%)	0 (0%)		1.0000
- Hematologic	6 (4.7%)	6 (6.5%)	0 (0%)		1.0000
- Mortality (30 days)	1 (0.8%)	1 (1.1%)	0 (0%)		1.0000
- Reoperation	17 (13.2%)	12 (12.9%)	5 (13.9%)		1.0000
Median overall survival (month)	29.17 (22.97 – 35.32)	34.99 (28.84 – 45.67)	19.88 (14.59 – 28.16)	–	**0.0028** [4]
Median DSS (month)	29.67 (25.20 – 37.19)	36.27 (29.17 – 47.08)	22.41 (18.92 – 29.60)	–	**0.0229** [4]

[1] TNM information was missing for 1-1 patient of both groups. [2] Results were available for a selected number of patients only. [3] Including primary tumor removal surgeries, diagnostic/explorative laparoscopies, metastasectomies. [4] *p*-Value acquired from Cox regression survival model. Ana: anastomosis; ASA: American Society of Anesthesiologists; CC: Sugarbaker's completeness of cytoreduction score; DSS: disease-specific survival; HIPEC: Hyperthermic Intraperitoneal Chemotherapy; ICU: intensive care unit; Perit: peritonectomy; sAC: synchronous PM + non-mucinous adenocarcinoma; sMAC: synchronous PM + mucinous adenocarcinoma.

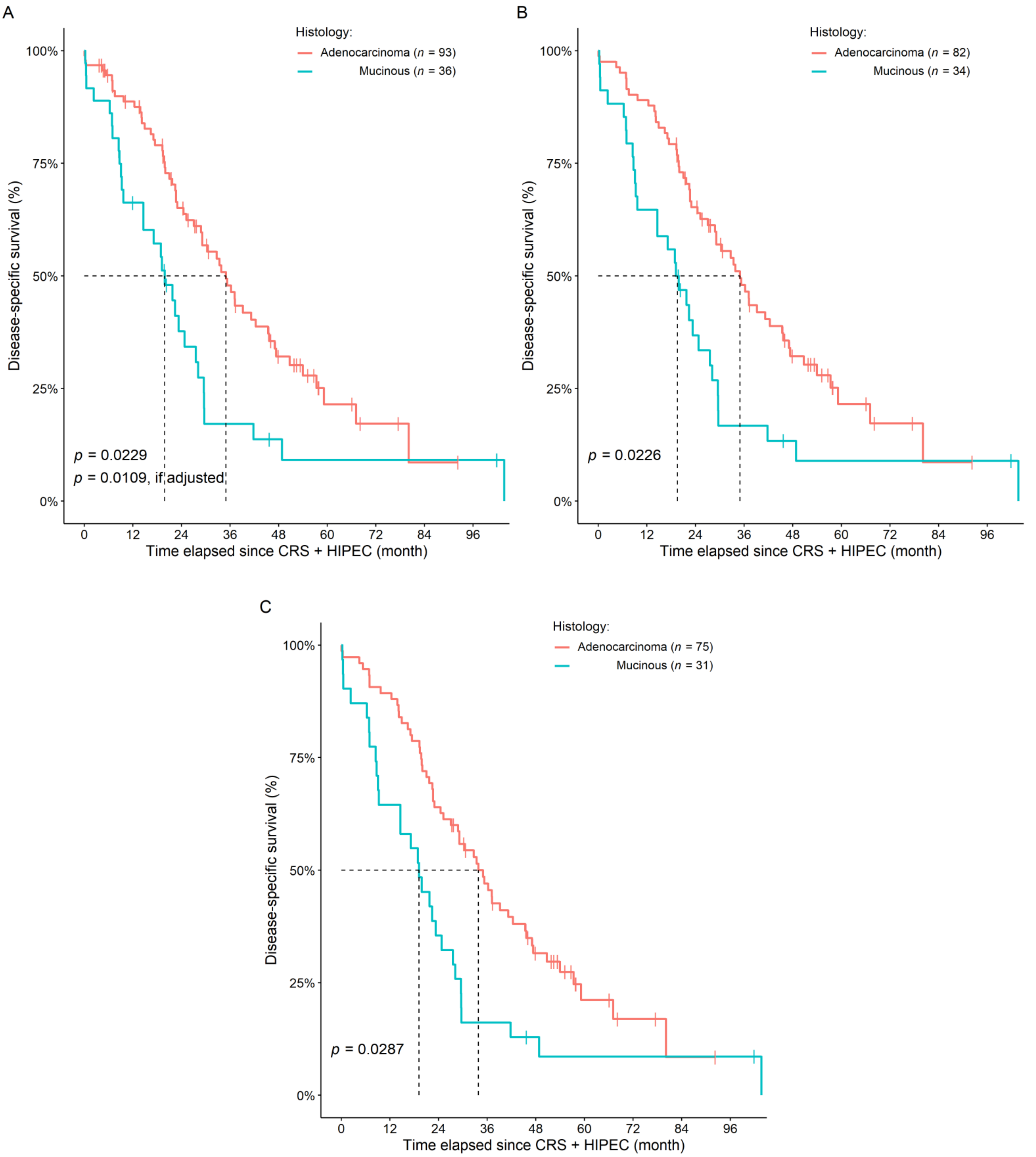

Figure S1. Differently adjusted survival curves of colorectal cancer patients with synchronous peritoneal metastasis. **A**: complete cohort; **B**: those patients were removed from the analysis, who had the CRS + HIPEC procedure in 2021; **C**: those patients were removed from the analysis, who had the CRS + HIPEC procedure in 202-2021. The histological diagnosis of mucinous adenocarcinoma was associated with inferior survival in all versions.

Table S2. Complete pre-, peri- and postoperative demographic and clinical characteristics of study participants having metachronous peritoneal metastases (PM). Unit of frequency and survival data is the number of observations (percentage) and median survival time (95% confidence interval), respectively.

Clinical Characteristics	Metachronous PM (*n* = 89)	mAC (*n* = 67)	mMAC (*n* = 22)	Crude *p*-value	Adjusted *p*-value
Age (year)	55.90 ± 11.76	56.27 ± 11.66	54.76 ± 12.26		1.0000
Body mass index (kg/m^2)	25.70 ± 4.93	25.05 ± 4.03	27.64 ± 6.72		1.0000
Sex (male:female)	45:44 (50.6%:49.4%)	35:32 (52.2%:47.8%)	10:12 (45.5%:54.5%)		1.0000
ASA score					1.0000
- I–II	20 (22.5%)	16 (23.9%)	4 (18.2%)		
- III–IV	69 (77.5%)	51 (76.1%)	18 (81.8%)		
Tumor location				**0.0064**	0.3991
- Cecum	13 (14.6%)	5 (7.5%)	8 (36.4%)		
- Ascending colon	13 (14.6%)	12 (17.9%)	1 (4.5%)		
- Hepatic flexure	5 (5.6%)	2 (3.0%)	3 (13.6%)		
- Transverse colon	3 (3.4%)	3 (4.5%)	0 (0%)		
- Splenic flexure	3 (3.4%)	3 (4.5%)	0 (0%)		
- Descending colon	7 (7.9%)	7 (10.4%)	0 (0%)		
- Sigmoid colon	28 (31.5%)	21 (31.3%)	7 (31.8%)		
- Rectosigmoid	2 (2.2%)	2 (3.0%)	0 (0%)		
- Rectum	14 (15.7%)	12 (17.9%)	2 (9.1%)		
- Unknown primary location	1 (1.1%)	0 (0%)	1 (4.5%)		
Sidedness				**0.0734**	1.0000
- Left-sided	45 (50.6%)	33 (49.3%)	7 (31.8%)		
- Right-sided	29 (32.6%)	22 (32.8%)	12 (54.5%)		
- Rectum	14 (15.7%)	12 (17.9%)	2 (9.1%)		
- Unknown primary location	1 (1.1%)	0 (0%)	1 (4.5%)		
Stage T (size of tumor) [1]					1.0000
- 1-2	2 (2.2%)	1 (1.5%)	1 (4.5%)		
- 3	51 (57.3%)	40 (59.7%)	11 (50.0%)		
- 4	32 (36.0%)	26 (38.8%)	7 (31.8%)		
Stage N (lymph node metastases) [1]					1.0000
- 0	23 (25.8%)	18 (26.9%)	5 (22.7%)		
- 1	36 (40.4%)	28 (41.8%)	6 (27.3%)		
- 2-3	28 (31.5%)	21 (31.3%)	8 (36.4%)		
Lineage of chemotherapy					1.0000
- Second line	54 (60.7%)	40 (59.7%)	14 (63.6%)		
- Third line	25 (28.1%)	21 (31.3%)	4 (18.2%)		
Usage of biological agents					1.0000
- Bevacizumab	16 (18.0%)	13 (19.4%)	3 (13.6%)		
- Cetuximab / Panitumumab	10 (11.2%)	7 (10.4%)	3 (13.6%)		
Radiotherapy	8 (9.0%)	8 (11.9%)	0 (0%)		1.0000
Any previous surgeries related to CRC [2]					
- Number of surgeries (single:multiple)	39:48 (43.8%:53.9%)	31:35 (46.3%:52.2%)	8:13 (36.4%:59.1%)		1.0000
- Primary tumor resecated	84 (94.4%)	65 (97.0%)	19 (86.4%)		1.0000
Type of previous surgeries [2]					
- Primary tumor resection only	49 (55.1%)	39 (58.2%)	10 (45.5%)		1.0000
- Laparoscopic exploration only	2 (2.2%)	1 (1.5%)	1 (4.5%)		1.0000
- Metastasis removal (excl. Perit.)	34 (38.2%)	24 (35.8%)	10 (45.5%)		1.0000
- Partial peritonectomy	8 (9.0%)	5 (7.5%)	3 (13.6%)		1.0000
Peritoneal carcinomatosis index	7.85 ± 5.70	7.43 ± 5.74	9.19 ± 5.49		1.0000

CC score					
- CC-0	78 (87.6%)	59 (88.1%)	19 (86.4%)		1.0000
- CC-1	10 (11.2%)	8 (11.9%)	2 (9.1%)		
- CC-2	1 (1.1%)	0 (0%)	1 (4.5%)		
Time between CRS+HIPEC and tumor diagnosis (month)	24.78 ± 20.07	23.52 ± 15.94	28.63 ± 29.47		1.0000
HIPEC medication					
- Oxaliplatin	50 (56.2%)	39 (58.2%)	11 (50.0%)		1.0000
- Mitomycin	39 (43.8%)	28 (41.8%)	11 (50.0%)		
Oxaliplatin (mg)	300.00 ± 20.20	297.44 ± 16.01	309.09 ± 30.15		1.0000
Mitomycin (mg)	26.67 ± 6.82	25.89 ± 6.67	28.64 ± 7.10		1.0000
Temperature of HIPEC					
- 41°C	10 (11.2%)	8 (11.9%)	2 (9.1%)		1.0000
- 42°C	79 (88.8%)	59 (88.1%)	20 (90.9%)		
Duration of HIPEC (min)					
- 30	50 (56.2%)	39 (58.2%)	11 (50.0%)		1.0000
- 60	17 (19.1%)	10 (14.9%)	7 (31.8%)		
- 90	22 (24.7%)	18 (26.9%)	4 (18.2%)		
Length of surgery excl. HIPEC (min)	283.3 ± 101.3	276.7 ± 100.7	303.4 ± 102.8		1.0000
Surgical procedures					
- Perit.: parietal	60 (67.4%)	44 (65.7%)	16 (72.7%)		1.0000
- Perit.: pelvis	58 (65.2%)	44 (65.7%)	14 (63.6%)		1.0000
- Perit.: omental bursa	4 (4.5%)	3 (4.5%)	1 (4.5%)		1.0000
- Perit.: right upper quadrant	25 (28.1%)	13 (19.4%)	12 (54.5%)	**0.0026**	0.1616
- Perit.: left upper quadrant	11 (12.4%)	5 (7.5%)	6 (27.3%)	**0.0238**	1.0000
- Ana.: small bowel–small bowel	26 (29.2%)	21 (31.3%)	5 (22.7%)		1.0000
- Ana.: stomach–small bowel	2 (2.2%)	2 (3.0%)	0 (0%)		1.0000
- Ana.: small bowel–colon	28 (31.5%)	18 (26.9%)	10 (45.5%)		1.0000
- Ana.: colon–colon	1 (1.1%)	1 (1.5%)	0 (0%)		1.0000
- Ana.: colon–rectum	35 (39.3%)	29 (43.3%)	6 (27.3%)		1.0000
- Ana.: small bowel–rectum	2 (2.2%)	1 (1.5%)	1 (4.5%)		1.0000
- Colostomy	9 (10.1%)	8 (11.9%)	1 (4.5%)		1.0000
- Ileostomy	15 (16.9%)	12 (17.9%)	3 (13.6%)		1.0000
- Colon resection	47 (52.8%)	32 (47.8%)	15 (68.2%)		1.0000
- Small bowel resection	37 (41.6%)	27 (40.3%)	10 (45.5%)		1.0000
- Rectosigmoid resection	41 (46.1%)	33 (49.3%)	8 (36.4%)		1.0000
- Splenectomy	10 (11.2%)	7 (10.4%)	3 (13.6%)		1.0000
- Cholecystectomy	44 (49.4%)	35 (52.2%)	9 (40.9%)		1.0000
- Bladder resection	4 (4.5%)	2 (3.0%)	2 (9.1%)		1.0000
- Greater omentectomy	67 (75.3%)	51 (76.1%)	16 (72.7%)		1.0000
- Lesser omentectomy	18 (20.2%)	14 (20.9%)	4 (18.2%)		1.0000
- Hepatic metastasectomy	10 (11.2%)	9 (13.4%)	1 (4.5%)		1.0000
- No. of resecated hepatic metastases	0.15 ± 0.58	0.18 ± 0.65	0.05 ± 0.21		1.0000
- Pancreas resection	4 (4.5%)	3 (4.5%)	1 (4.5%)		
- Stomach resection	1 (1.1%)	1 (1.5%)	0 (0%)		1.0000
- Hysterectomy	22 (24.7%)	15 (22.4%)	7 (31.8%)		1.0000
- Adnexectomy	29 (32.6%)	21 (31.3%)	8 (36.4%)		1.0000
- Resection of other organs	34 (38.2%)	26 (38.8%)	8 (36.4%)		1.0000
Length of hospital stay (day)	17.06 ± 7.19	16.37 ± 6.72	19.24 ± 8.32		1.0000
Length of ICU stay (day)	3.84 ± 2.16	3.70 ± 2.01	4.27 ± 2.57		1.0000
Complication grade (Clavien–Dindo)					1.0000
- I	38 (42.7%)	31 (46.3%)	7 (31.8%)		

- II	29 (32.6%)	21 (31.3%)	8 (36.4%)		
- III	22 (24.7%)	15 (22.4%)	7 (31.8%)		
Complications					
- Anastomotic insufficiency/leak	8 (9.0%)	7 (10.4%)	1 (4.5%)		1.0000
- Pancreatitis	1 (1.1%)	1 (1.5%)	0 (0%)		1.0000
- Fistula	3 (3.4%)	3 (4.5%)	0 (0%)		1.0000
- Pulmonary embolism	1 (1.1%)	1 (1.5%)	0 (0%)		1.0000
- Hematologic	5 (5.6%)	3 (4.5%)	2 (9.1%)		1.0000
- Mortality (30 days)	0 (0.0%)	0 (0%)	0 (0%)		1.0000
- Reoperation	12 (13.5%)	9 (13.4%)	3 (13.6%)		1.0000
Median overall survival (month)	32.10 (26.97 – 45.40)	32.10 (27.93 – 45.44)	26.18 (19.12 – NA) [3]	–	0.8210 [4]
Median DSS (month)	34.43 (28.48 – 48.13)	34.43 (29.96 – 48.13)	32.56 (19.12 – NA) [3]	–	0.6490 [4]

[1] TNM information was missing for 3 patients of the mucinous group due to the fact that no primary tumor resection was performed. [2] Including primary tumor removal surgeries, diagnostic/explorative laparoscopies, metastasectomies. [3] Not enough observation to reach lower 95% confidence interval. [4] *p*-Value acquired from Cox regression survival model. Ana: anastomosis; ASA: American Society of Anesthesiologists; CC: Sugarbaker's completeness of cytoreduction score; DSS: disease-specific survival; HIPEC: Hyperthermic Intraperitoneal Chemotherapy; ICU: intensive care unit; mAC: metachronous PM + non-mucinous adenocarcinoma; mMAC: metachronous PM + mucinous adenocarcinoma; Perit: peritonectomy.

Table S3. Comparison results of all four study groups (adjusted *p*-values of post-hoc test). The corresponding count and numerical data can be seen in Tables S1 and S2.

Clinical parameter	mAC vs. mMAC	mAC vs. sAC	mAC vs. sMAC	mMAC vs. sAC	mMAC vs. sMAC	sAC vs. sMAC
Age	0.9549	0.8529	0.5200	1.0000	0.9388	0.8601
Body mass index	0.1317	0.9335	0.9788	0.2518	0.1046	0.8022
1.0000Sex	1.0000	1.0000	0.9585	1.0000	1.0000	1.0000
ASA score	1.0000	1.0000	1.0000	0.8236	1.0000	0.9360
Tumor location	0.8318	0.6371	0.1008	0.4228	0.2175	0.5136
Sidedness	0.1940	0.2115	**0.0159**	0.8095	0.6757	0.3403
Stage T (size of tumor)	0.7517	**0.0006**	**< 0.0001**	0.0995	**0.0116**	0.2322
Stage N (lymph node metastases)	1.0000	**0.0476**	0.0827	0.8844	0.9658	0.7302
Lineage of chemotherapy	1.0000	0.0557	0.7560	1.0000	1.0000	0.3621
Usage of biological agents	0.9767	0.1593	0.4790	0.5046	0.5825	1.0000
Radiotherapy	0.3781	0.9503	0.9487	0.8935	0.8644	0.8614
Any previous surgeries related to CRC	0.6927	**0.0328**	**0.0085**	0.4578	0.1509	0.5140
- Primary tumor resecated	0.2684	**0.0027**	**0.0003**	0.4643	0.1359	0.4165
- 1.0000	1.0000	0.9234	1.0000	0.8670	0.6877	1.0000
- Partial peritonectomy	1.0000	**0.0024**	0.9118	0.3507	1.0000	**0.0278**
Peritoneal carcinomatosis index	0.6569	0.8710	0.1241	0.3277	0.9265	**0.0189**
CC score	1.0000	0.8922	1.0000	1.0000	1.0000	0.9283
Time between CRS+HIPEC and tumor diagnosis (month)	0.5035	**< 0.0001**	**0.0001**	**< 0.0001**	**< 0.0001**	1.0000
HIPEC medication	1.0000	1.0000	0.9903	1.0000	1.0000	1.0000
Temperature of HIPEC	1.0000	0.9818	1.0000	1.0000	1.0000	1.0000
Duration of HIPEC (min)	1.0000	1.0000	1.0000	1.0000	0.9947	1.0000
Length of surgery excl. HIPEC (min)	0.7163	0.8655	0.9946	0.3713	0.8678	0.8079
Surgical procedures						
- Perit.: parietal	1.0000	1.0000	1.0000	1.0000	0.7989	1.0000
- Perit.: pelvis	0.8645	1.0000	1.0000	1.0000	1.0000	1.0000
- Perit.: omental bursa	0.9910	1.0000	0.0818	1.0000	0.2732	0.0621
- Perit.: right upper quadrant	**0.0099**	0.4643	0.0709	**0.0282**	0.5898	0.1727
- Perit.: left upper quadrant	0.0921	0.6968	**0.0025**	0.1092	1.0000	**0.0034**

Surgical procedures						
- Ana.: small bowel–small bowel	1.0000	1.0000	0.2968	0.9274	0.8950	1.0000
- Ana.: stomach–small bowel	1.0000	0.6638	1.0000	1.0000	1.0000	1.0000
- Ana.: small bowel–colon	0.4872	0.8894	0.9858	0.4165	1.0000	1.0000
- Ana.: colon–colon	1.0000	0.2284	1.0000	0.3682	1.0000	0.2512
- Ana.: colon–rectum	0.9298	0.5623	1.0000	0.9824	0.5294	0.9720
- Ana.: small bowel–rectum	1.0000	0.8162	0.3660	1.0000	1.0000	0.3733
- Colostomy	0.6806	**0.0120**	0.2164	1.0000	0.7659	1.0000
- Ileostomy	1.0000	1.0000	0.8779	1.0000	1.0000	1.0000
- Colon resection	0.3888	0.4681	0.9217	0.1629	0.9353	0.4774
- Small bowel resection	1.0000	0.9401	0.8910	1.0000	1.0000	0.8914
- Rectosigmoid resection	1.0000	0.2577	1.0000	0.7945	1.0000	1.0000
- Splenectomy	1.0000	0.5545	0.8613	0.4815	0.6920	0.0926
- Cholecystectomy	1.0000	1.0000	1.0000	1.0000	1.0000	0.9927
- Bladder resection	0.5187	0.7508	1.0000	0.3711	0.2430	1.0000
- Greater omentectomy	1.0000	1.0000	1.0000	1.0000	1.0000	0.9045
- Lesser omentectomy	0.8009	1.0000	1.0000	1.0000	1.0000	1.0000
- Hepatic metastasectomy	0.9598	0.6639	0.7574	0.2950	1.0000	0.7268
- No. of resecated hepatic metastases	0.8484	0.5406	0.8998	0.3040	0.9967	0.2680
- Pancreas resection	1.0000	0.2267	0.5361	0.6163	0.6354	1.0000
- Stomach resection	1.0000	1.0000	0.3689	1.0000	0.3936	0.0921
- Hysterectomy	1.0000	0.7718	1.0000	1.0000	1.0000	1.0000
- Adnexectomy	0.6529	1.0000	1.0000	1.0000	1.0000	1.0000
- Resection of other organs	0.8284	0.3325	0.4003	0.8537	1.0000	1.0000
Length of hospital stay (day)	0.6879	0.9978	0.9789	0.7395	0.8940	0.9935
Length of ICU stay (day)	0.8173	0.6850	0.9708	0.9986	0.9681	0.9714
Complication grade (Clavien–Dindo)	0.6142	0.5244	0.4534	0.1173	0.1901	0.9680

Complications						
- Anastomotic insufficiency/leak	1.0000	0.7110	1.0000	0.9665	1.0000	1.0000
- Pancreatitis	1.0000	1.0000	1.0000	1.0000	1.0000	0.8319
- Fistula	0.8777	0.2267	1.0000	1.0000	1.0000	1.0000
- Pulmonary embolism	1.0000	1.0000	1.0000	1.0000	1.0000	1.0000
- Hematologic	1.0000	1.0000	1.0000	0.6119	0.7543	0.6705
- Mortality (30 days)	1.0000	1.0000	1.0000	1.0000	1.0000	1.0000
- Reoperation	0.	0.	0.	0.	0.	0.
Median overall survival (month)	0.5966	0.6899	**0.0118**	0.7861	**0.0179**	**0.0031**
Median DSS (month)	0.7728	0.9686	**0.0302**	0.7879	0.0782	**0.0239**

mAC: metachronous peritoneal metastasis + non-mucinous adenocarcinoma; mMAC: metachronous peritoneal metastasis + mucinous adenocarcinoma; sAC: synchronous peri-toneal metastasis + non-mucinous adenocarcinoma; sMAC: synchronous peritoneal metastasis + mucinous adenocarcinoma.
